# Proteomics reveals the function reverse of MPSSS‐treated prostate cancer‐associated fibroblasts to suppress PC‐3 cell viability via the FoxO pathway

**DOI:** 10.1002/cam4.3825

**Published:** 2021-03-11

**Authors:** Tingting Zhang, Xiulan Chen, Lang Sun, Xiaojing Guo, Tanxi Cai, Jifeng Wang, Yanqiong Zeng, Jing Ma, Xiang Ding, Zhensheng Xie, Lili Niu, Mengmeng Zhang, Ning Tao, Fuquan Yang

**Affiliations:** ^1^ Key Laboratory of Protein and Peptide Pharmaceuticals & Laboratory of Proteomics Institute of Biophysics, Chinese Academy of Sciences Beijing China; ^2^ University of Chinese Academy of Sciences Beijing China; ^3^ School of Basic Medical Sciences of Southwest Medical University Luzhou China

**Keywords:** cell cycle arrest, MPSSS, quantitative proteomics, TGF‐β3, the FoxO pathway

## Abstract

Prostate cancer‐associated fibroblasts (prostate CAFs) are essential components of the tumor microenvironment and can promote tumor progression through their immunosuppressive functions. MPSSS, a novel polysaccharide purified from *Lentinus edodes*, has been reported to have anti‐tumor activity. MPSSS could also inhibit the immunosuppressive function of prostate CAFs, which has been demonstrated through that the secretome of MPSSS‐treated prostate CAFs could inhibit the proliferation of T cells. However, how the secretome of MPSSS‐treated prostate CAFs influence prostate cancer progression is still unclear. Interestingly, we found that the low molecular weight (3–100kD) secretome of prostate CAFs (lmwCAFS) could promote the growth of PC‐3 cells, while that of MPSSS‐treated prostate CAFs (MT‐lmwCAFS) could inhibit their growth. We carried out comparative secretomic analysis of lmwCAFS and MT‐lmwCAFS to identify functional molecules that inhibit the growth of PC‐3 cells, and proteomic analysis of lmwCAFS‐treated PC‐3 cells and MT‐lmwCAFS‐treated PC‐3 cells to investigate the underlying molecular mechanism. These analyses suggest that TGF‐β3 from MT‐lmwCAFS may inhibit the growth of PC‐3 cells. The validated experiments revealed that TGF‐β3 from MT‐lmwCAFS activated p21 expression in PC‐3 cells by regulating the FoxO pathway thereby inducing G0/G1 cell cycle arrest of PC‐3 cells. Overall, our data demonstrated that MPSSS reversed the ability of prostate CAFs to suppress the cell viability of PC‐3 cells, which might provide a potential therapeutic strategy to prevent prostate cancer progression.

## Graphical abstract

MPSSS reversed the tumor‐promoting effect of prostate CAFs; TGF‐β3 was upregulated in MPSSS‐treated prostate CAFs to arrest the PC‐3 cells in G0/G1 phase via the FoxO pathway.

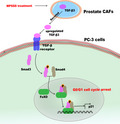

## 1 INTRODUCTION

Prostate cancer (PCa) remains the malignancy with the highest morbidity in men.^1^ Although early‐stage PCa usually causes no symptoms, cancer cells in prostate glands readily metastasize to bones, lymph nodes, lungs, and liver during PCa progression.^2^ Common strategies, including surgery, radiation therapy, and androgen‐deprivation therapy (ADT), are promising treatments for early‐stage PCa. However, advanced PCa still lacks effective therapeutic strategies.^3^, ^4^, ^5^ Thus, researchers are currently focused on inhibiting tumor progression to explore effective therapeutic strategies for PCa treatment.^6^, ^7^

Cancer‐associated fibroblasts (CAFs), stromal cells derived from normal fibroblasts or epithelia, are one of the major cellular components in the tumor microenvironment.^8^, ^9^ Compared with normal fibroblasts, CAFs are activated with increased expression of key protein markers, such as α‐SMA, FAP, vimentin, and S100A4 protein.^10^, ^11^ In the tumor microenvironment, CAFs secrete various growth effectors or cytokines to the extracellular matrix and thereby promote tumor progression by directly enhancing cancer cell growth, angiogenic induction, the extracellular matrix modification, and tumor‐promoting inflammatory mediation.^11^, ^12^, ^13^, ^14^ Notably, CAFs could act as an immunosuppressive mediator for the formation of an immunosuppressive tumor environment by recruiting and activating multiple immune cells.^15^ As such, CAFs might be a potential target in anti‐tumor immunotherapy.^16^, ^17^ Currently, increased researches have focused on inhibiting the immunosuppressive function of PCa‐associated fibroblasts (prostate CAFs), which may suggest therapeutic strategies for PCa.^18^, ^19^

Polysaccharides from *Lentinus edodes* are known to exhibit potent anti‐tumor and immunomodulatory functions. The anti‐tumor mechanisms of *L*.* edodes* polysaccharides include regulating the innate immune system by mediating CAF function, preventing tumorigenesis, or directly killing tumor cells via cell cycle regulation or apoptotic induction.^20^, ^21^, ^22^ Our previous studies showed that a novel polysaccharide component (MPSSS) purified from *L*.* edodes* could inhibit tumor growth in vivo. Briefly, C57BL/6 mice were subcutaneously injected with McgR32 cells and then randomly separated into two groups. Eight days later, the test group and the control group were intraperitoneally injected with MPSSS (25 mg/kg) and PBS respectively once two days for eight days. The tumors from the MPSSS‐treated group grew slower and were significantly smaller than those in the control group.^23^ MPSSS could also prevent the immunosuppressive function of prostate CAFs, and that the secretome of prostate CAFs treated with MPSSS could inhibit the proliferation of CD4^+^ and CD8^+^ T cells.^22^ However, how the secretome of MPSSS‐treated prostate CAFs affect PCa progression is still unclear.

The secretome defines as all proteins secreted by the organism or living cells into the extracellular space, which consists of soluble proteins and extracellular vesicles (EVs).^24^ The secretome of prostate CAFs pays vital roles in PCa tumor origination and progression.^25^, ^26^ Since EVs contains various molecules (proteins, RNA, DNA and lipid),^27^ we speculate that soluble proteins and EVs of prostate CAFs might have the different influence on PCa cells. The fractionation could reduce the range of active components and is beneficial to the discovery of active molecules. In this study, the secretome of prostate CAFs untreated/treated with MPSSS was separated into the high molecular weight secretome (>100 kD), and the low molecular weight secretome (3–100 kD) using 100‐kD and 3‐kD MWCO membrane filtration to narrow down the range of active components. The high molecular weight secretome contained EVs and large soluble proteins, while the low molecular weight secretome contained the small soluble proteins. The secretome of prostate CAFs untreated/treated with MPSSS was used to explore the underlying function and molecular mechanism using quantitative proteomics and multiple biochemical approaches.

## 2 MATERIALS AND METHODS

### 2.1 Cell culture

Prostate CAFs were kindly provided by Professor Ju Zhang at the College of Life Sciences, Nankai University. The human PCa cell line PC‐3 was kindly provided by Professor Weiqiang Gao of School of biomedical engineering, Shanghai Jiao Tong University. All cell lines were cultured in DMEM media (CM15019, Macgene Biotech, Beijing, China) supplemented with 10% FBS 100 mg/ml of streptomycin and 100 U/ml of penicillin at 37°C and 5% CO_2_.

### 2.2 The preparation of MPSSS

Crude polysaccharides from *L*.* edodes* were purchased from Johncan International in Hangzhou, China. MPSSS was purified as described in the previous studies.^22^, ^23^

### 2.3 The preparation of CM from prostate CAFs treated with different concentrations of MPSSS

The procedure for preparing conditioned medium (CM) from prostate CAFs with different concentrations of MPSSS is shown in Figure S1. CM^0^, CM^0.2^, CM^0.4^, CM^0.6^, CM^0.8^, and CM^1.0^ were harvested.

### 2.4 Fractionation of the supernatants of prostate CAFs untreated/treated with MPSSS

**FIGURE 1:**
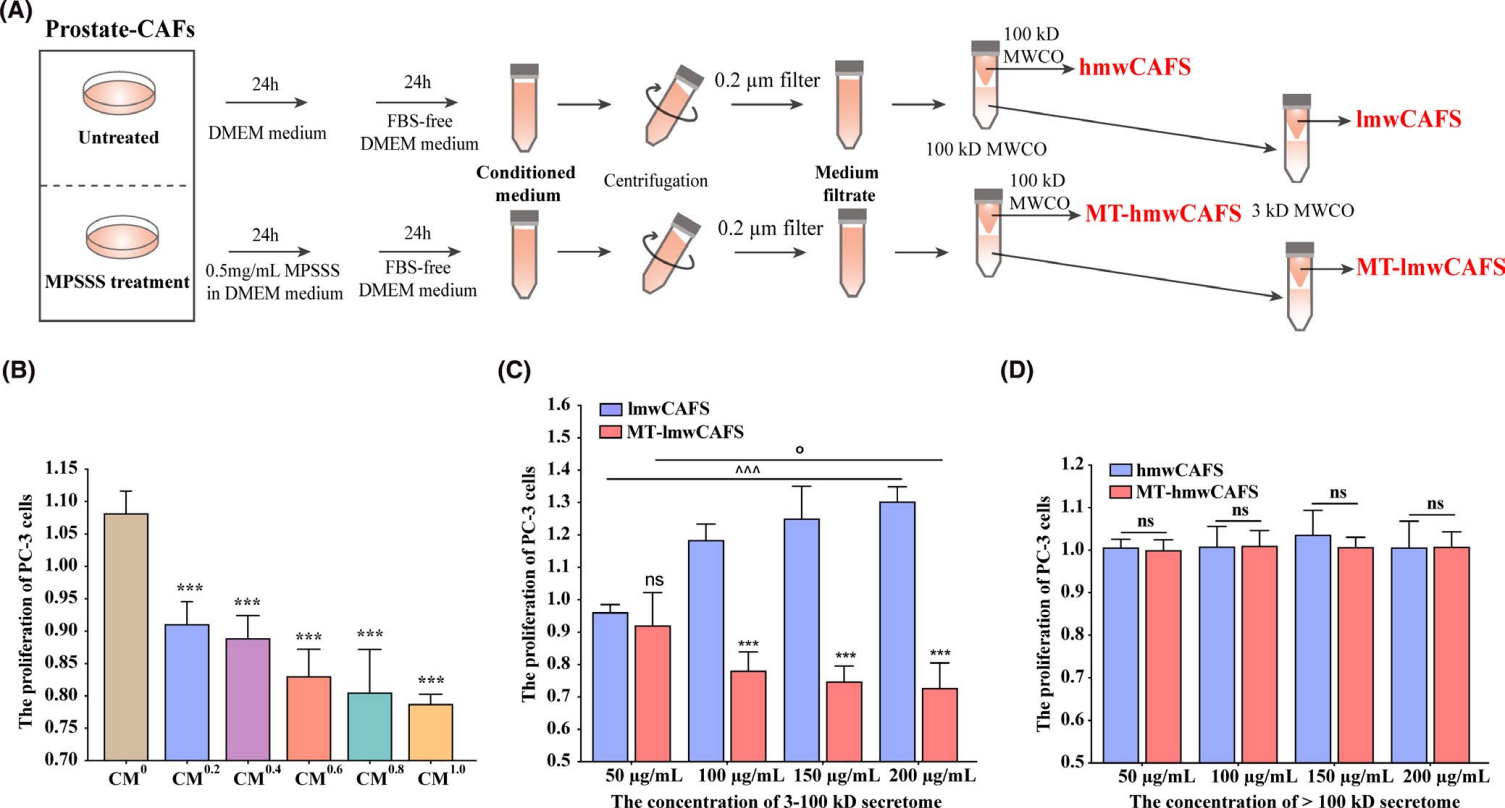
The effect of lmwCAFS and MT‐lmwCAFS on PC‐3 cells. (A) The workflow for collecting high molecular weight and low molecular weight secretome of prostate CAFs untreated/treated with MPSSS. (B) PC‐3 cells were exposed to CM^0^, CM^0.2^, CM^0.4^, CM^0.6^, CM^0.8^, and CM^1.0^ and fresh DMEM (v/v = 1:1) for 24 h. *n* = 4 biological replicates, ***p ≤ 0.001: PC‐3 cells treated with CM^0.2^, CM^0.4^, CM^0.6^, CM^0.8^, and CM^1.0^ compared with PC‐3 cells treated with CM^0^, respectively. (C) PC‐3 cells were exposed to lmwCAFS and MT‐lmwCAFS (3‐ to 100‐kD secretome) with final protein concentrations of 50, 100, 150, and 200 μg/ml for 24 h. *n* = 4 biological replicates, ns and ***p ≤ 0.001: PC‐3 cells treated with MT‐lmwCAFS compared with PC‐3 cells treated with lmwCAFS. ^^^^^ *p* ≤0.001: PC‐3 cells treated with lmwCAFS. °*p* ≤0.001: PC‐3 cells treated with MT‐lmwCAFS. (D) PC‐3 cells were exposed to hmwCAFS and MT‐hmwCAFS (>100‐kD secretome) at final protein concentrations of 50, 100, 150, and 200 μg/ml for 24 h

Our previous study showed that MPSSS reduced prostate CAFs activity by decreasing α‐SMA expression, and 0.5 mg/ml of MPSSS obviously decreased the expression.^22^ In this study, α‐SMA level was measured in prostate CAFs untreated and treated with 0.5 mg/ml of MPSSS. The result showed that the expression of α‐SMA in prostate CAFs was decreased with the treatment of 0.5 mg/ml of MPSSS (Figure S2). Therefore, 0.5 mg/ml of MPSSS was chosen to treat prostate CAFs in this study. Prostate CAFs were untreated/treated with MPSSS for 24 h and then cultured in FBS‐free DMEM for another 24 h. CM was harvested, centrifuged at 1000 *g* for 3 min followed by 2000 *g* for 20 min, and filtered using a 0.2‐μm filter (PALL) to remove dead cells and cell debris. To explore the functional molecules on PC‐3 cells, the supernatants of prostate CAFs untreated/treated with MPSSS were separated into the high molecular weight secretome (>100 kD) (hmwCAFS/MT‐hmwCAFS) and the low molecular weight secretome (<100 kD) with Amicon Ultra‐15 tubes (100‐kD MWCO; Millipore). The low molecular weight secretome (<100 kD) was then concentrated with Amicon Ultra‐4 tubes (3 kD MWCO; Millipore) to produce the low molecular weight secretome (3–100 kD) samples (lmwCAFS/MT‐lmwCAFS) (Figure 1A). The protein concentrations of all fractions were determined using BCA assay (Thermo Fisher, USA).

### 2.5 Cell viability

3000–4000 cells were seeded in 96‐well plates per well and incubated with conditioned media for 24 h. MTT assays were used to measure cell viability with an EnSpire multifunction microplate reader (Perkin Elmer). Triplicate samples were used for each group in all experiments. Student's *t* test was used for comparisons between two groups, and one‐way ANOVA was used for comparisons among three or more groups.

### 2.6 TMT 6‐plex labeling

Proteins in lmwCAFS/MT‐lmwCAFS were solubilized in lysis buffer (8‐M urea, 100‐mM HEPES, pH 8.5). PC‐3 cells treated with lmwCAFS/MT‐lmwCAFS were lysed in lysis buffer (8‐M urea, 100‐mM HEPES, pH 8.5). The protein mixtures were digested using mass spectrometry grade Lys‐C (Wako) and modified trypsin (Promega) as previously described.^28^ Equal amounts of peptides were labeled with TMT 6‐plex reagents according to the manufacturer's protocols. Then, the labeled peptides were pooled, dried and resolved with 100‐mM HEPES buffer, followed by desalting with an Oasis HLB column (Waters) and drying for high pH fractionation.

### 2.7 High pH reversed‐phase fractionation

The peptides were dissolved in buffer A (2% ACN/98% H_2_O, pH = 10) and fractioned on a RIGO 3000 equipped with an XBridge peptide BEH C18 column (130 Å, 3.5 μm, 2.1 × 100 mm, Waters) as described in a previous study.^28^ Thirteen fractions were collected for LC‐MS/MS analysis.

### 2.8 LC‐MS/MS analysis

Peptide samples (900 ng for each) were reconstituted in 0.1% FA/H_2_O and analyzed by LC‐MS/MS with an Easy n‐LC 1000 HPLC and a Q Exactive mass spectrometer (Thermo Fisher Scientific, USA). The peptides were separated with a C18 column (75 µm × 20 cm) packed with Reprosil‐Pur C18 AQ particles (3.0 μm, Dr. Maisch HPLC GmbH) with solvent A (0.1% FA) and solvent B (ACN/0.1% FA) at a flow rate of 280 nl/min with a gradient: 4% B (0 min), 8% B (5 min), 22% (58 min), 32% B (70 min), 90% B (71 min), and 90% B (78 min).

A Q Exactive mass spectrometer (Thermo Fisher Scientific, USA) was used for MS analysis in data‐dependent acquisition mode. Each MS1 spectrum was obtained at 70,000 high‐resolution (m/z 200) at 300–1600 m/z. The automatic gain control (AGC) target value was 3E6 for a maximum filling time of 60 ms. The top 20 most abundant precursor ions were selected with a 2.0‐m/z isolation window and fragmented with a normalized collision energy of 27. MS/MS spectra were acquired at 17,500 resolution (m/z 200) with a 50,000 target value over a maximum injection time of 80 ms by setting up an isolation window of 2.0 m/z and dynamic exclusion time of 40 s.

### 2.9 Protein identification and quantification

The raw data were processed using Proteome Discoverer software v2.2 with Sequest HT and Mascot search engine using a human database (including 20,385 proteins downloaded in August, 2018) appended with known contaminants. Trypsin was selected as the enzyme, and only two missed cleavages were allowed. The precursor mass tolerance was 10 ppm, and the product ion tolerance was 0.02 Da. Carbamidomethyl cysteine and TMT 6‐plex labeled lysine(K) and N‐terminus of peptides were selected as fixed modifications, while methionine oxidation and N‐terminal acetylation were set as variable modifications. The percolator algorithm was used for FDR analysis. Peptides with FDR <1% were set as high confidence peptides.

### 2.10 Bioinformatics analysis

R (v3.6.0) was used for statistical analysis performed by Student's *t* test with Benjamin–Hochberg adjustment. The genuine‐secreted proteins were predicted with SignalP,^29^ SecretomeP,^30^ and UniProt.^31^, ^32^ GO, KEGG pathway and STRING analyses were used to analyze the differentially expressed proteins.

### 2.11 qRT‐PCR analysis

Total RNA was extracted from cells with TRIzol reagent (Invitrogen, Carlsbad, CA, USA). A 5× All‐In‐One RT MasterMix (abmGood, Canada) was used for reverse transcription to synthesize cDNA. Then, the mRNA level of p21 and GAPDH was quantified using SYBR Premix ExTaq (TaKaRa, Tokyo, Japan) with the 2^−ΔΔCt^ method according to the manufacturer's instructions. The primer sequences are shown in Table S1.^33^, ^34^, ^35^

### 2.12 Small interfering RNA

Three pairs of siRNAs for TGFBR2, FoXO3, and p21, and the negative control siRNA were acquired from GenePharma (Shanghai, China). The siRNA sequences are shown in Table S2; 100 nM of siRNAs were transfected into PC‐3 cells using Lipofectamine 3000 (Invitrogen) according to the manufacturer's protocol. Then, the knockdown efficiencies were determined using western blot analysis. The results showed that knockdown efficiencies for TGFBR2‐siRNA1, TGFBR2‐siRNA3, FoXO3‐siRNA2, FoXO3‐siRNA3, p21‐siRNA1, and p21‐siRNA3 are very effective (Figure S3). Therefore, these siRNAs were chosen for subsequent experiments.

### 2.13 Coimmunoprecipitation analysis

PC‐3 cell lysates were added to smad3 (9513, CST) and FoxO3 antibodies (99199, CST), and incubated overnight at 4°C with gentle shaking. Protein A/G magnetic beads (Thermo Fisher Scientific) were added and incubated at 4°C for 2 h. Subsequently, the sample was analyzed by Western blotting.

### 2.14 Western blotting analysis

The western blotting analysis was performed as previously described.^36^ The primary antibodies were against TGF‐β3 (5344R, BioVision), p21 (ab109520, Abcam), Smad4 (sc‐73040, Santa), and Smad4 (ab40759, Abcam); HRP‐conjugated primary antibody against GAPDH was used as the loading control. HRP‐conjugated goat anti‐rabbit IgG and anti‐mouse IgG was used as the secondary antibody.

### 2.15 Cell cycle assay

Cells for cell cycle analysis were stained with propidium iodide (PI). The stained cells were analyzed by flow cytometer (BD FACSCalibur), and collected data were analyzed using FlowJo software.

## 3 RESULTS

### 3.1 MPSSS reverses the ability of prostate CAFs to inhibit the growth of PC‐3 cells

To explore how the secretome of MPSSS‐treated prostate CAFs affect PCa cells, we collected CM from prostate CAFs treated with gradient MPSSS (CM^0^, CM^0.2^, CM^0.4^, CM^0.6^, CM^0.8^, CM^1.0^). Consistent with studies showing that prostate CAFs contribute to tumor development,^12^ CM^0^ showed to promote the growth of PC‐3 cells (Figure 1B). However, CM^0.2^, CM^0.4^, CM^0.6^, CM^0.8^, and CM^1.0^ inhibited PC‐3 proliferation, and the inhibitory effects became more significant as the concentration of MPSSS increased (Figure 1B). To further explore which secreted molecules in secretome of prostate CAFs displayed growth‐regulatory effects on PC‐3 cells, we narrowed the CM of prostate CAFs untreated/treated with MPSSS down the high molecular weight secretome (hmwCAFS/MT‐hmwCAFS) and the low molecular weight secretome (lmwCAFS/MT‐lmwCAFS). When PC‐3 cells were treated with lmwCAFS and MT‐lmwCAFS, lmwCAFS promoted the growth of PC‐3 cells, while MT‐lmwCAFS significantly prevented their growth (Figure 1C). The promoting effect of lmwCAFS and inhibitory effect of MT‐lmwCAFS became more significant as the increase of their final concentration of proteins (Figure 1C). However, no obvious effects were found in PC‐3 cells treated with hmwCAFS and MT‐hmwCAFS (Figure 1D). Overall, MPSSS reversed the ability of prostate CAFs thereby inhibit the proliferation of PC‐3 cells.

### 3.2 Quantitative proteomic analyses of lmwCAFS/MT‐lmwCAFS and lmwCAFS‐treated PC‐3 cells/MT‐lmwCAFS‐treated PC‐3 cells

**FIGURE 2:**
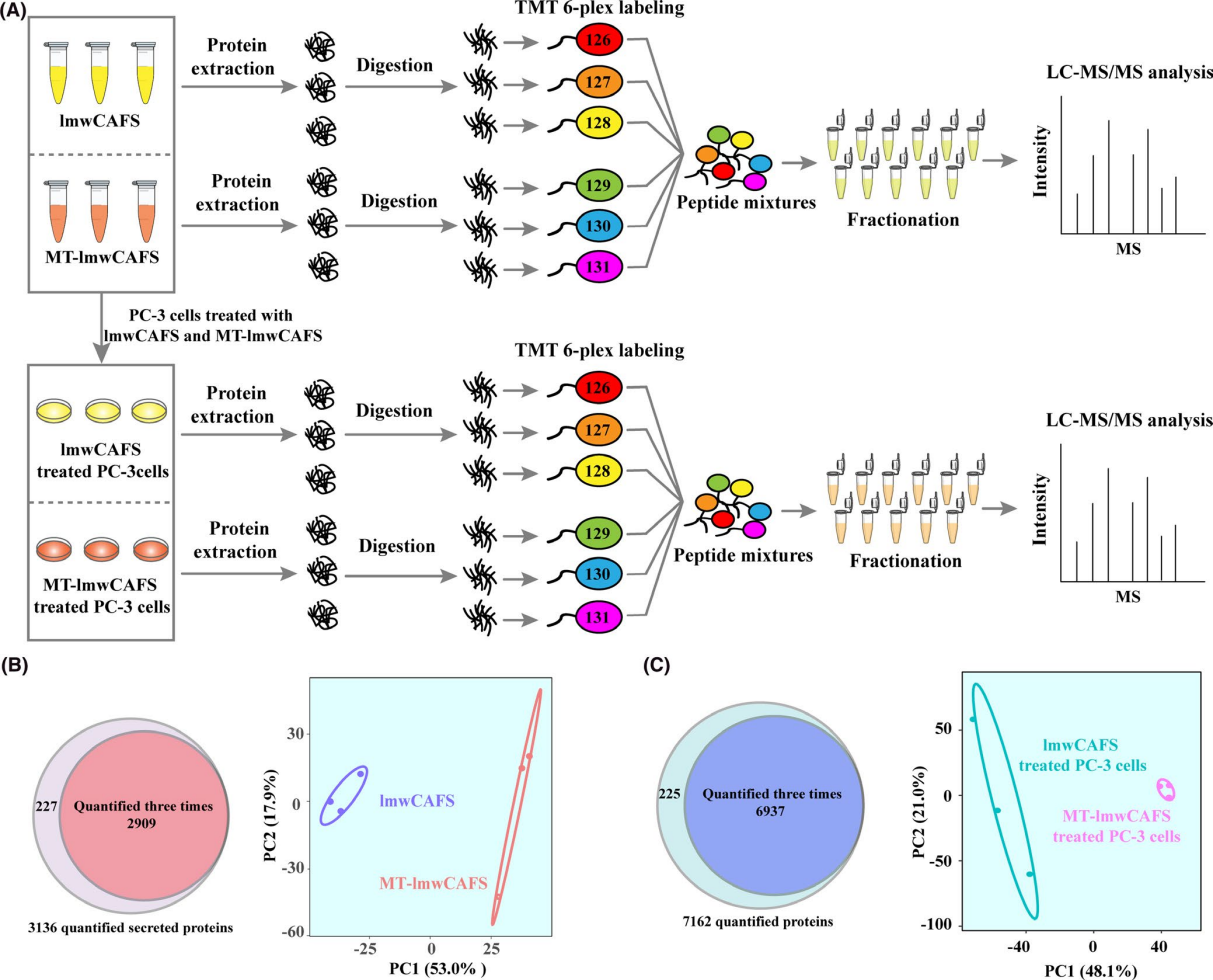
Quantitative analysis of the secretome and proteome using TMT labeling. (A) Schematic workflow of secretomic analysis of lmwCAFS/MT‐lmwCAFS and proteomic analysis of lmwCAFS‐treated PC‐3 cells/MT‐lmwCAFS‐treated PC‐3 cells. PC‐3 cells were treated with lmwCAFS/MT‐lmwCAFS at a final concentration of 200 μg/ml for 24 h. (B) Quantified numbers of lmwCAFS/MT‐lmwCAFS protein groups, and (C) of lmwCAFS‐treated PC‐3 cells/MT‐lmwCAFS‐treated PC‐3 cells are showed using a venn diagram (left) and principal component analysis of proteins quantified by three times (right)

To explore how MPSSS‐treated prostate CAFs inhibit the growth of PC‐3 cells, we carried out comparative secretomic analysis of lmwCAFS and MT‐lmwCAFS to identify functional molecules that inhibit the growth of PC‐3 cells. We also performed proteomic analysis of lmwCAFS‐treated PC‐3 cells and MT‐lmwCAFS‐treated PC‐3 cells to investigate the underlying mechanism. Three biological replicates of lmwCAFS/MT‐lmwCAFS‐ and lmwCAFS‐treated PC‐3 cells/MT‐lmwCAFS‐treated PC‐3 cells were labeled with TMT 6‐plex and analyzed by LC‐MS/MS (Figure 2A). There were 2909 protein groups quantified three times in lmwCAFS/MT‐lmwCAFS (Figure 2B, left) and 6937 protein groups quantified three times in lmwCAFS‐treated PC‐3 cells/MT‐lmwCAFS‐treated PC‐3 cells (Figure 2C, left). Principal component analysis showed that the secretome differed between lmwCAFS and MT‐lmwCAFS, and the proteome differed between lmwCAFS‐treated PC‐3 cells and MT‐lmwCAFS‐treated PC‐3 cells, respectively. Three biological replicates had good reproducibility (Figure 2B, right and Figure 2C, right).

### 3.3 The prediction of genuine‐secreted proteins in lmwCAFS and MT‐lmwCAFS

**FIGURE 3:**
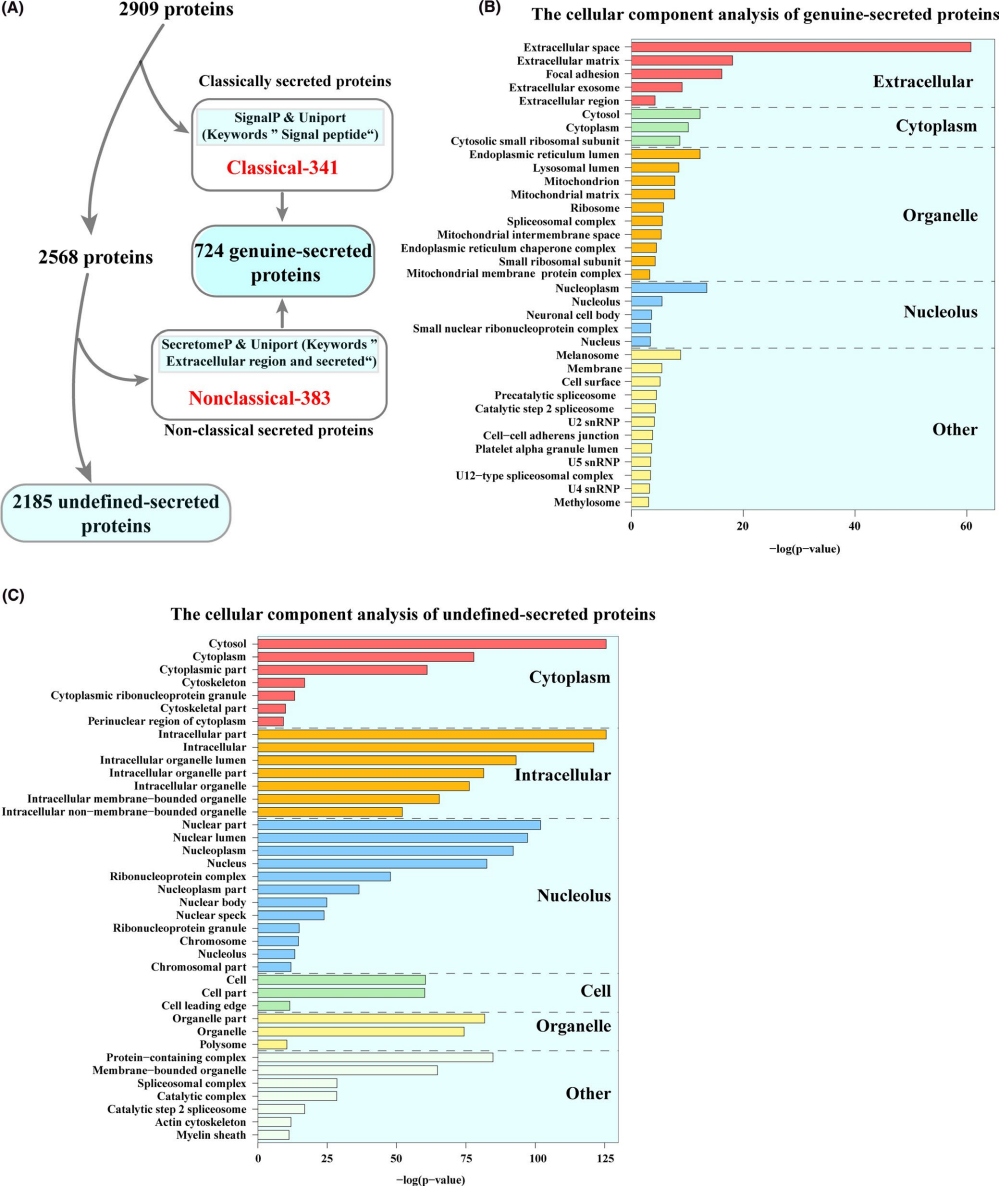
The prediction and cellular component enrichment of secreted proteins. (A) The workflow of genuine‐secreted proteins predicted by combining different bioinformatic tools. (B) The cellular component analysis of genuine‐secreted proteins. (C) The cellular component analysis of undefined‐secreted proteins

The secretome contains proteins with signal peptides secreted through ER−Golgi transport, and proteins located in extracellular regions released through different nonclassical pathways.^31^, ^37^ In secretome analysis of CM, many detected proteins are not defined as part of the secretome. These proteins might be secreted through nonclassical pathways but not defined as secreted proteins, or from shedding and rupture cells because of cells’ overwhelming abundance of cultured cells.^32^ To distinguish genuine‐secreted proteins and undefined‐secreted proteins, we used a combination of SignalP, SecretomeP, and UniProt with the keyword annotations—“Signal” and “Extracellular and secreted” to analyze the identification data. With these bioinformatics methods, 724 of 2909 (24.9%) proteins were predicted as genuine‐secreted proteins including 341 classical and 383 nonclassical secreted proteins (Figure 3A and Table S3). This percentage of genuine‐secreted proteins from lmwCAFS/MT‐lmwCAFS is consistent with the previous studies.^31^, ^32^ The cellular component analyses indicated that most of the genuine‐secreted proteins were enriched in extracellular (Figure 3B) and that the other 2185 proteins, which were considered as undefined‐secreted proteins, were mostly enriched in cytoplasm and intracellular (Figure 3C). After molecular function analysis of the differentially expressed proteins in the undefined‐secreted proteins, no potential proteins related to cell growth regulation were found (Figure S4). Thus, only the genuine‐secreted proteins were used for subsequent analysis and validation.

### 3.4 The bioinformatic analysis of differentially expressed proteins in lmwCAFS/MT‐lmwCAFS and lmwCAFS‐treated PC‐3 cells/MT‐lmwCAFS‐treated PC‐3 cells

**FIGURE 4:**
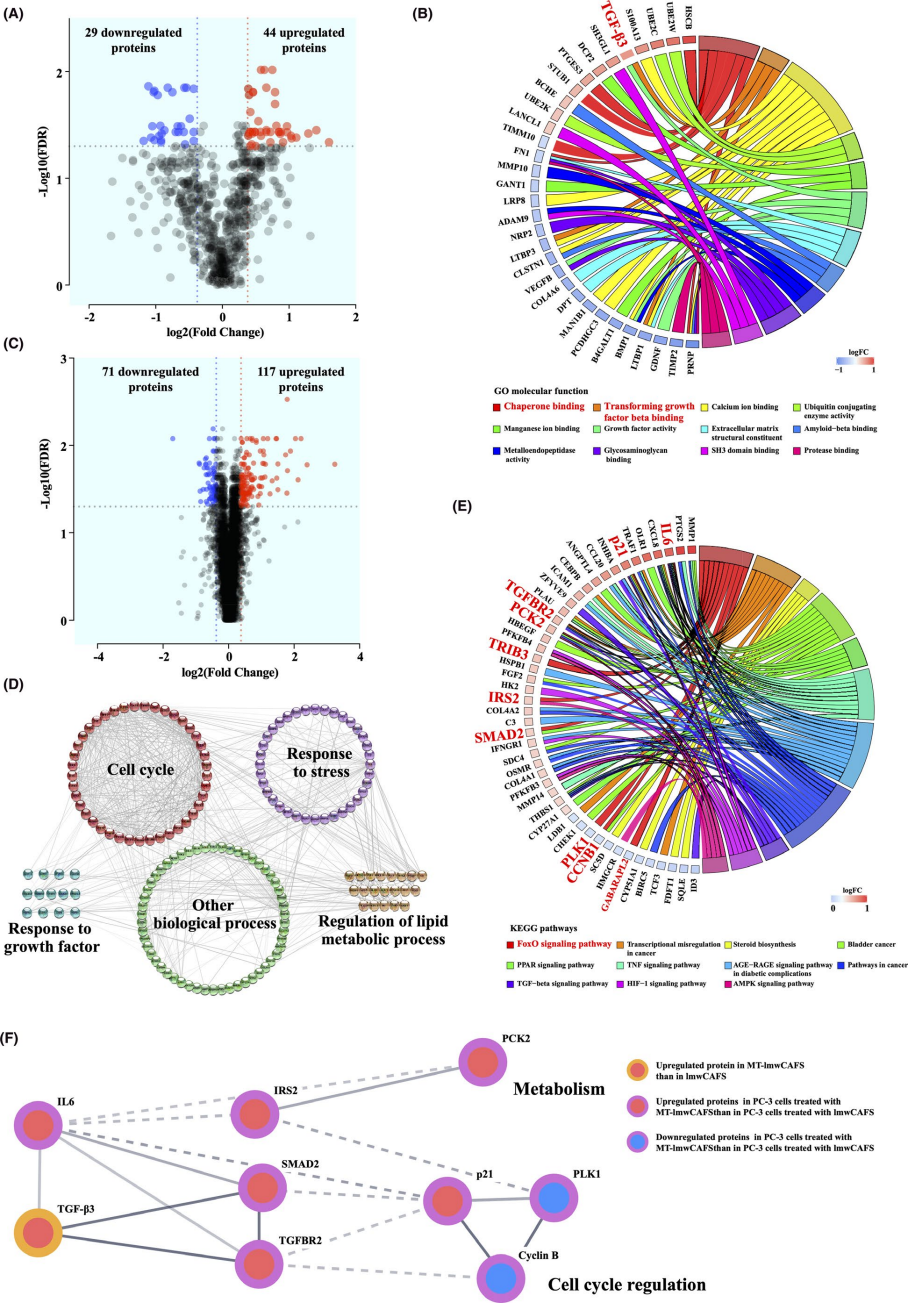
The bioinformatic analysis of differentially expressed proteins. (A) Volcano plots showing the differentially expressed proteins in lmwCAFS and MT‐lmwCAFS. (B) The molecular function analysis of differentially expressed proteins in lmwCAFS and MT‐lmwCAFS shown in the chord plot. (C) Volcano plots showing the differentially expressed proteins in lmwCAFS‐treated PC‐3 cells/MT‐lmwCAFS‐treated PC‐3 cells. (D) Biological process analysis of the differentially expressed proteins in lmwCAFS‐treated PC‐3 cells/MT‐lmwCAFS‐treated PC‐3 cells. (E) KEGG pathway analysis of the differentially expressed proteins in lmwCAFS‐treated PC‐3 cells/MT‐lmwCAFS‐treated PC‐3 cells shown in the chord plot. The red fonts represent proteins belonging to the FoxO pathway. (F) Protein interactions in the FoxO pathway. In the chord plot, proteins are linked with their assigned terms via ribbons. Red‐to‐blue rectangles next to selected proteins represent their logFC. GO molecular function or KEGG pathway terms are arranged from top to bottom according to their significance

We analyzed significantly differential expression proteins in the lmwCAFS/MT‐lmwCAFS and lmwCAFS treated PC‐3 cells/MT‐lmwCAFS treated PC‐3 cells. For 724 genuine‐secreted proteins, 73 proteins were significantly differential expressed with a *p* value <0.05 and a fold‐change score of >1.3 or <0.77 (Table S4). Among them, 44 proteins were upregulated, and 29 proteins were downregulated in MT‐lmwCAFS compared to lmwCAFS (Figure 4A). The heatmap analysis of the 73 differential expressional proteins showed that lmwCAFS and MT‐lmwCAFS could cluster and easily distinguish the differential expression of proteins (Figure S5A). These differentially expressed proteins were enriched for chaperone binding and transforming growth factor beta (TGF‐β) binding. HscB and TGF‐β3 are highly differentially expressed proteins in chaperone binding and transforming growth factor beta binding, respectively (Figure 4B). HscB acts as a cochaperone of Hsp70 and mediates iron‐sulfur cluster biogenesis.^38^ It has been reported that highly expressed TGF‐β3 is tightly linked to PCa suppression.^39^ TGF‐β3 was strongly upregulated in MT‐lmwCAFS compared to lmwCAFS. These results suggested that TGF‐β3 from MT‐lmwCAFS might be a vital molecule to inhibit PC‐3 cell proliferation.

In the lmwCAFS‐treated PC‐3 cells and MT‐lmwCAFS‐treated PC‐3 cells, 188 proteins were the differential expression, including 71 downregulated proteins and 117 upregulated proteins (*p* value <0.05 and fold change >1.3 or <0.77) (Figure 4C, Table S5). The heatmap analysis of these 188 proteins showed that PC‐3 cells treated with lmwCAFS and MT‐lmwCAFS well cluster and easily distinguish the differential expression of proteins (Figure S5B). The biological process analysis showed that these differentially expressed proteins were enriched in the cell cycle, regulation of lipid metabolic, response to stress, and response to growth factor (Figure 4D). Among them, cell cycle was the most prominent biological process (Figure S5C). The KEGG pathway analysis highlighted that the Forkhead box O (FoxO) signaling pathway was the most remarkable pathway (Figure 4E). The FoxO pathway is regulated by the TGF‐β, insulin, and AMPK signaling,^40^, ^41^, ^42^ and the aforementioned results suggested that TGF‐β3 from MT‐lmwCAFS might inhibit PC‐3 cell proliferation. Therefore, we hypothesized that TGF‐β3 from MT‐lmwCAFS potentially interacts with proteins belonging to the FoxO pathway in PC‐3 cells. To validate this hypothesis, TGF‐β3 and proteins belonging to the FoxO pathway were combined, and integrated analysis was performed using the STRING database.^43^ These proteins were significantly enriched in the FoxO pathway, and we found that TGF‐β3 directly interacted with IL‐6, SMAD2, and TGFBR2 (TGF beta receptor 2) (Figure 4F). TGF‐β was reported to directly interact with TGFBR2 to induce Smad‐dependent signaling in the FoxO pathway.^44^ In addition, p21, Plk1, and cyclin B were directly interacted, while IRS2 directly interacted with PCK2 (Figure 4F). PCK2 was reported to be tightly associated with glycolysis,^45^ whereas few studies revealed that PCK2 upregulation could inhibit cell proliferation. p21 (cyclin‐dependent kinase inhibitor 1) potently inhibits the activity of the cyclin B‐CDK complex (M‐phase‐promoting factor); thus, a high level of p21 arrests the cell cycle in G1 phase.^46^, ^47^ Although Plk1 could trigger the G2/M transition by activating the cyclin B‐CDK complex,^48^ p21 was significantly upregulated, and cyclin B and Plk1 were both downregulated in MT‐lmwCAFS‐treated PC‐3 cells compared to lmwCAFS treated PC‐3 cells (Figure 4F). According to these analyses, we speculate that TGF‐β3 from MT‐lmwCAFS might arrest the cell cycle progression of PC‐3 cells and thereby to inhibit their growth.

### 3.5 TGF‐β3 from MT‐lmwCAFS could induce G1 cell cycle arrest of PC‐3 cells

**FIGURE 5:**
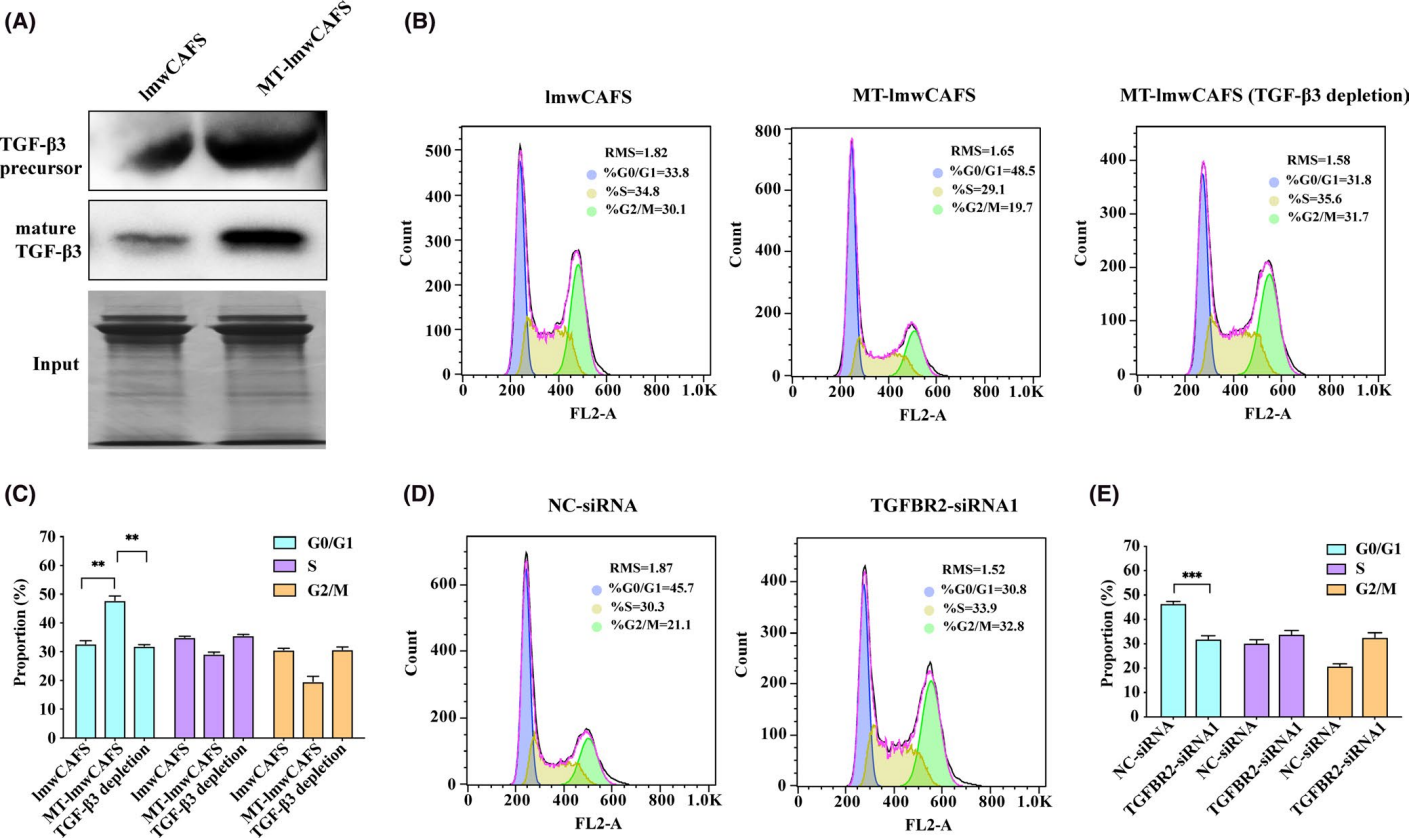
TGF‐β3 from MT‐lmwCAFS led to cell cycle arrest of PC‐3 cells. (A) Western blotting of TGF‐β3 in lmwCAFS and MT‐lmwCAFS. SDS‐PAGE of lmwCAFS and MT‐lmwCAFS was performed as input. (B) and (C) Cell cycle analysis of the PC‐3 cells treated with lmwCAFS, MT‐lmwCAFS and MT‐lmwCAFS with TGF‐β3 ligand immune depletion. (D) and (E) Cell cycle analysis of the PC‐3 cells transfected with TGFBR2 siRNA1 (TGFBR2‐siRNA1) or negative control siRNA (NC‐siRNA) and treated with MT‐lmwCAFS

To test the aforementioned hypothesis, we firstly validated the secretomic results by measuring the TGF‐β3 protein levels in lmwCAFS and MT‐lmwCAFS using western blotting. The results showed that TGF‐β3 was significantly upregulated in MT‐lmwCAFS compared to lmwCAFS, consistent with the LC‐MS/MS results (Figure 5A). The flow cytometry was used to analyze the cell cycle distribution of PC‐3 cells with different treatments. The cell cycle results showed that the percentage of PC‐3 cells in the GO/G1 phase after treatment with MT‐lmwCAFS was significantly higher than that of the lmwCAFS group (Figure 5B and C). Then TGF‐β3 was blocked signaling by either TGF‐β3 ligand immune depletion from MT‐lmwCAFS or TGFBR2 knockdown in PC‐3 cells treated with MT‐lmwCAFS. The flow cytometry analysis of cell cycle distribution showed that TGF‐β3 depletion led to attenuation of MT‐lmwCAFS induced G0/G1 cell cycle arrest (Figure 5B and C), and TGFBR2 knockdown also resulted in attenuation of MT‐lmwCAFS induced G0/G1 cell cycle arrest compared to negative control siRNA transfected PC‐3 cells (Figure 5D and E). These results suggested TGF‐β3 form MT‐lmwCAFS arrest PC‐3 cells in G0/G1 phase.

### 3.6 TGF‐β3 from MT‐lmwCAFS‐induced G0/G1 cell cycle arrest of PC‐3 cells via the FoxO pathway

**FIGURE 6:**
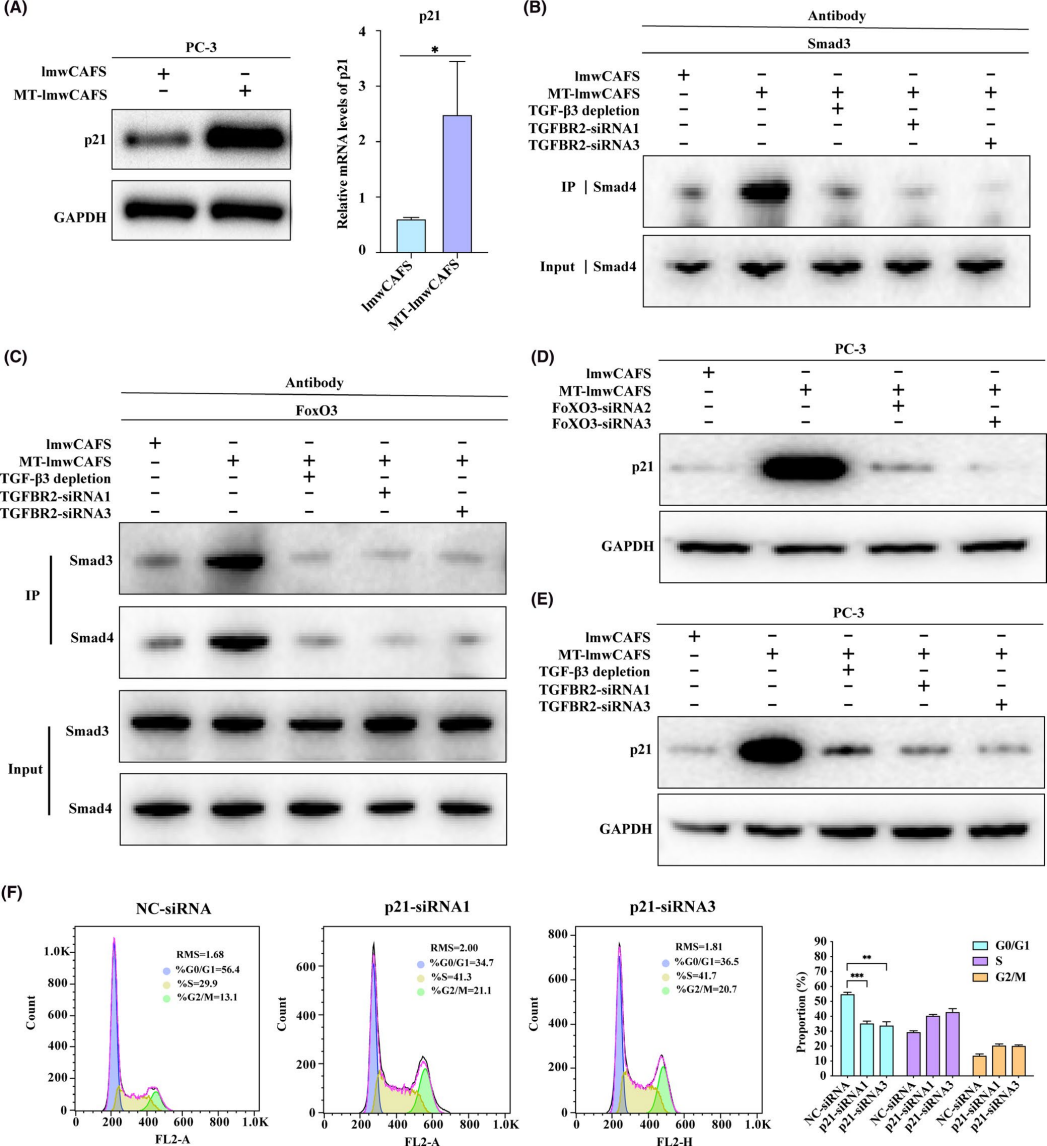
TGF‐β3 from MT‐lmwCAFS activated the FoxO pathway in PC‐3 cells. (A) Western‐blotting and RT‐PCR of p21 in lmwCAFS‐treated PC‐3 cells/MT‐lmwCAFS‐treated PC‐3 cells. (B) and (C) Coimmunoprecipitation analysis of the PC‐3 cells treated with lmwCAFS, MT‐lmwCAFS and MT‐lmwCAFS with TGF‐β3 ligand immune depletion, and the PC‐3 cells with TGFBR2 knockdown and MT‐lmwCAFS treatment. (D) Western blotting of p21 in the PC‐3 cells treated with lmwCAFS and MT‐lmwCAFS, and the PC‐3 cells with FoxO3 knockdown and MT‐lmwCAFS treatment. (E) Western blotting of p21 in the PC‐3 cells treated with lmwCAFS, MT‐lmwCAFS, and MT‐lmwCAFS with TGF‐β3 ligand immune depletion, and the PC‐3 cells with TGFBR2 knockdown and MT‐lmwCAFS treatment. (F) Cell cycle analysis of the PC‐3 cells transfected with p21 siRNA (p21‐siRNA1 and p21‐siRNA3) or negative control siRNA (NC‐siRNA) and treated with MT‐lmwCAFS

p21, as a cell cycle regulator, could trigger G0/G1 cell cycle arrest.^46^, ^47^ Seoane even reported that TGF‐β promotes the expression of p21 by regulating FoxO signaling.^49^ We hypothesized that TGF‐β3 from prostate CAFs may affect the expression of p21 and thereby regulate the cell cycle of PC‐3 cells via the FoxO pathway. To demonstrate this hypothesis, we validated p21 expression in PC‐3 cells treated with lmwCAFS/MT‐lmwCAFS using western blotting and qRT‐PCR. Consistent with the proteomic data, these results showed that the expression of p21 was markedly increased in the MT‐lmwCAFS‐treated PC‐3 cells compared to the lmwCAFS‐treated PC‐3 cells (Figure 6A). Coimmunoprecipitation experiments in PC‐3 cells showed that the smad3–smad4 complex was activated by MT‐lmwCAFS and interacted with the FoxO3 protein (Figure 6B and C). Subsequently, we revealed that FoxO3 was essential for p21 induction by knocking down of FoxO3 by FoxO3‐siRNA2 and FoXO3‐siRNA3 in MT‐lmwCAFS‐treated PC‐3 cells (Figure 6D). When TGF‐β3 signal was blocked by TGF‐β3 ligand immune depletion from MT‐lmwCAFS or TGFBR2 knockdown by TGFBR2‐siRNA1 and TGFBR2‐siRNA3 in PC‐3 cells, the results showed that TGF‐β3 from MT‐lmwCAFS was required for p21 activation via the formation of the smad‐FoxO complex (Figure 6B, C and E). The flow cytometry analysis of cell cycle distribution showed that p21 knockdown led to attenuation of MT‐lmwCAFS‐induced G0/G1 cell cycle arrest compared to negative control siRNA transfected PC‐3 cells, which was approved through p21 knockdown by p21‐siRNA1 and p21‐siRNA3 in MT‐lmwCAFS treated PC‐3 cells. These results suggested that TGF‐β3 from MT‐lmwCAFS regulated p21 expression in PC‐3 cells via the FoxO pathway, thereby arresting the cell cycle in G0/G1 phase.

## 4 DISCUSSION

**FIGURE 7:**
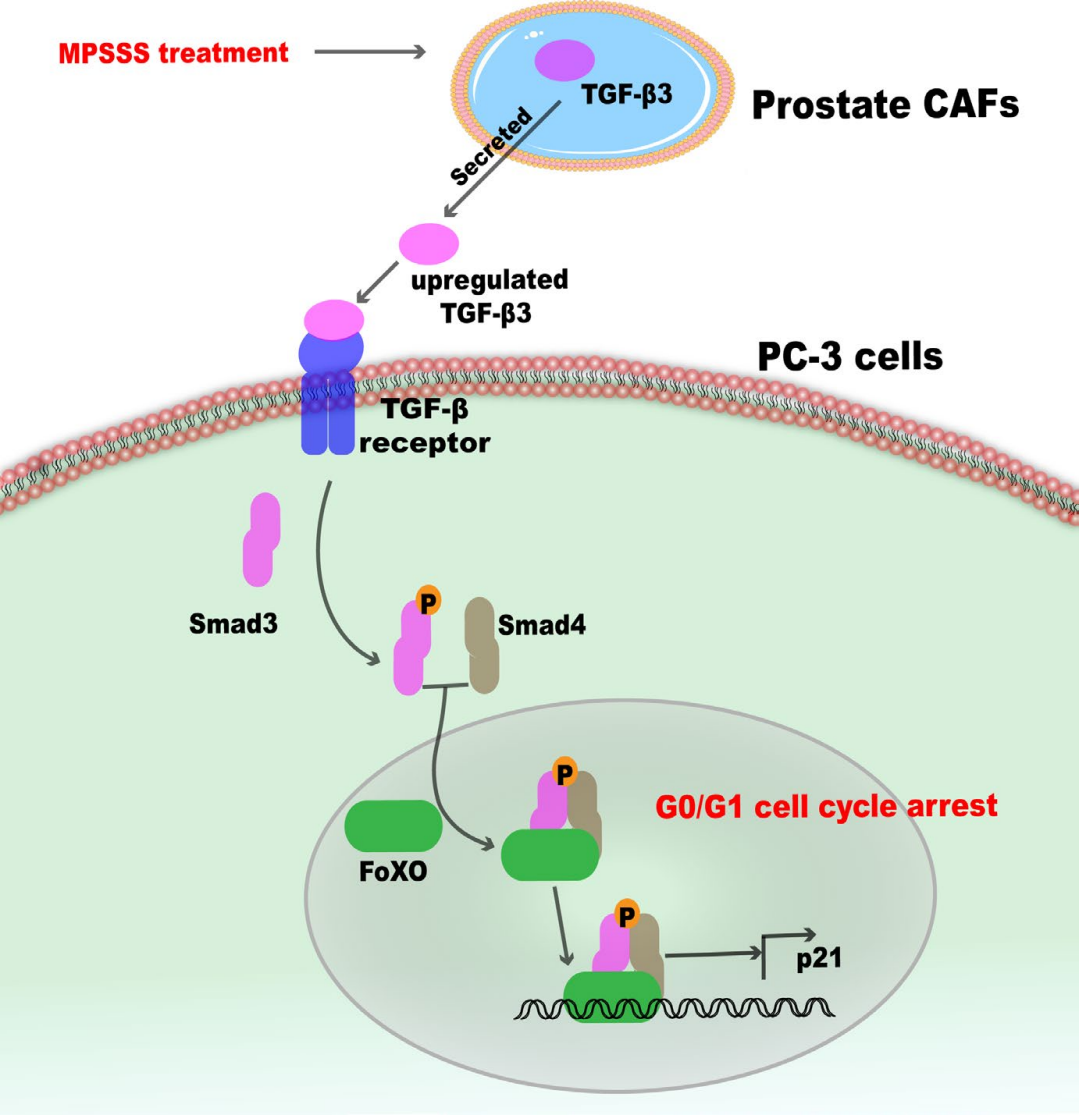
Model of TGF‐β3 from prostate CAFs with MPSSS treatment regulating the cell cycle of PC‐3 cells. TGF‐β3 from MPSSS‐treated prostate CAFs is upregulated and leads to the formation of the smad3—smad4 complex. This complex interacts with FoxO protein to activate p21 and arrest the PC‐3 cells in G0/G1 phase

In this study, we found that the effect of prostate CAFs was reversed by MPSSS, thereby inhibiting the proliferation of PC‐3 cells. Quantitative proteomic analyses of lmwCAFS/MT‐lmwCAFS‐ and lmwCAFS‐treated PC‐3 cells/MT‐lmwCAFS‐treated PC‐3 cells and multiple validation experiments revealed that TGF‐β3 was upregulated in MPSSS‐treated prostate CAFs and thereby activated p21 expression, arresting the PC‐3 cells in G0/G1 phase via the FoxO pathway (Figure 7).

As the soil of PCa cells in the tumor microenvironment, prostate CAFs promoted the proliferation of PC‐3 cells, whereas MPSSS reversed the tumor‐promoting effect of prostate CAFs and inhibited the growth of PC‐3 cells. Consistent with our study, it has been reported that reversing prostate CAFs functions via CXCL12 downregulation suppressed PCa progression.^50^ The quantitative proteomic analysis revealed that upregulated TGF‐β3 from MPSSS‐treated prostate CAFs might mediate the inhibition of PC‐3 cell proliferation. The TGF‐β family contains three similar forms, TGF‐β1, TGF‐β2, and TGF‐β3, which act as efficient tumor suppressors on premalignant cells or potent tumor promoters on cancerous cells.^51^ TGF‐β1 derived from CAFs has been reported to promote PCa cell growth and metastasis.^25^ Largely contrasting with this study, we found that MPSSS increased TGF‐β3 expression in prostate CAFs and thereby induced cell cycle arrest of PC‐3 cells, which was consistent with the studies showing that upregulating the expression of TGF‐β was relevant to tumor cell cycle arrest. For instance, ellagic acid led to cell cycle arrest of breast cancer cells via the TGF‐β pathway, ginsenoside Rh2 promoted TGF‐β expression to induce cell cycle arrest of leukemia cells, and pirfenidone significantly increased TGF‐β secretion and induced cell cycle arrest of PCa cells.^52^, ^53^, ^54^

p21 in PC‐3 cells was significantly increased by TGF‐β3 from MT‐lmwCAFS. In agreement with previous research,^55^ upregulated p21 induced PC‐3 cell cycle arrest in G0/G1 phase. Moreover, we first reported that TGF‐β3 from MT‐lmwCAFS activated smad proteins to form a complex with FoxO3, thereby regulating p21 expression in PC‐3 cells, while TGF‐β has been demonstrated to act as a FoxO signaling regulator, activating p21 expression in epithelial cells by regulating the formation of the smad‐FoxO complex.^49^, ^56^ Since highly activating the PI3 K‐Akt pathway and Forkhead box protein G1 (FoxG1) in tumors prevented the formation of the smad‐FoxO complex, which both led to TGF‐β unable to activate p21 expression, we speculated that some functional proteins from MT‐lmwCAFS might impede the PI3 K‐Akt pathway or FoxG1 expression and found that TGF‐β3 activates p21 via the FoxO pathway. However, this speculation needs to be confirmed in the future.

The fractionation could reduce the range of active components and is beneficial to the discovery of active molecules. Thus, we separated the secretome of prostate CAFs untreated/treated with MPSSS into high molecular weight secretome (containing EVs) and the low molecular weight secretome (just containing soluble proteins) and explored their influence on PCa cells. We interestingly found that the low molecular weight secretome could regulate the proliferation of PC‐3 cells, while the high molecular weight secretome (containing EVs) did not display this function. So, in this study, we just focused on how the low molecular weight secretome affect the proliferation of PC‐3 cells using comparative proteomics. Since EVs were the important components of the secretome and the researches have revealed that EVs derived from CAFs could mediated the proliferation, migration, and invasion of cancer cells,^57^, ^58^, ^59^ the high molecular weight secretome might have other unknown influence on PC‐3 cells, which need to be further studied.

TGF‐β can be activated and released through integrin mediation and proteases degrading LAP.^60^ In addition, Forkhead Box A1 (FOXA1) could directly regulate TGF‐β3 expression in PCa cells.^61^ However, it is still unclear how MPSSS promotes TGF‐β3 secretion in prostate CAFs. Although ADT is a common PCa treatment, many patients progress to become castration‐resistant prostate cancer (CRPC) after treatment with ADT for some time.^62^ As a CRPC cell line, PC‐3 is commonly used to explore potential therapies for CRPC patients.^61^, ^62^ Our data preliminarily revealed that TGF‐β3 from MT‐lmwCAFS induced p21 expression to prevent the PC‐3 cell cycle via the FoxO pathway. However, how MT‐lmwCAFS acts on other CRPC cell lines should be further studied.

In summary, through a comprehensive proteomics and biochemical analysis, we found that MPSSS reversed the tumor‐promoting effect of prostate CAFs cells by upregulating TGF‐β3, inducing cell cycle arrest via the FoxO pathway. Our findings first reveal that polysaccharides from *L*.* edodes* (MPSSS) could reverse the tumor‐promoting effect of prostate CAFs to suppress the cell viability of PC‐3 cells, which would contribute to the development of therapeutic strategies that effectively prevent PCa progression.

## CONFLICT OF INTEREST

The authors declare no conflict of interest.

## ETHICAL APPROVAL

Ethical approval may not need for this study. Because all experiments didn't involve animals or clinical samples in this study.

## Supporting information

Fig S1‐S5

Table S1

Table S2

Table S3

Table S4

Table S5

## Data Availability

The proteomics data are available in Proteomics Identification Database (PRIDE). The accession number is PXD023689.
