# Supplementary material for: Proteomics reveals the function reverse of MPSSS‐treated prostate cancer‐associated fibroblasts to suppress PC‐3 cell viability via the FoxO pathway
Source: Cancer Med. 2021 Mar 11;10(7):2509–22. doi: 10.1002/cam4.3825 (PMC7982613; doi:10.1002/cam4.3825)
Supplement: Supplementary file 1 — Fig S1‐S5 [file CAM4-10-2509-s005.docx]

Figure. S1.


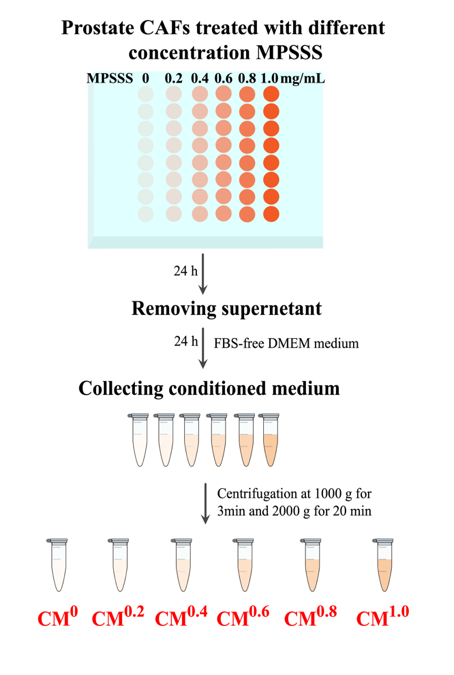


Figure. S1. The workflow of prostate-CAFs conditioned medium. Prostate-CAFs were seeded in 96-well plate with 3,000 – 4,000 cells per well, and treated with gradient MPSSS (0, 0.2 mg/mL, 0.4 mg/mL, 0.6 mg/mL, 0.8 mg/mL and 1 mg/mL) for 24 h. After that, MPSSS was removed and replaced with fresh DMEM medium to culture for another 24 h. Subsequently, conditioned medium was harvested, centrifuged at a low speed (1000 g for 3 min and 2000 g for 20 min) to remove dead cells and cell debris. The conditioned medium (CM) from prostate-CAFs untreated/treated with gradient MPSSS used as CM^0^, CM^0.2^, CM^0.4^, CM^0.6^, CM^0.8^, CM^1.0^.

Figure. S2.


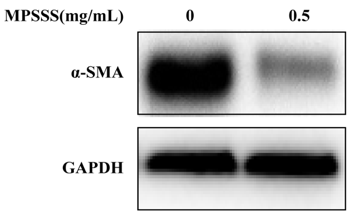


Figure. S2. Western blotting of α-SMA in the prostate CAFs untreated and treated with 0.5 mg/mL MPSSS.

Figure. S3.


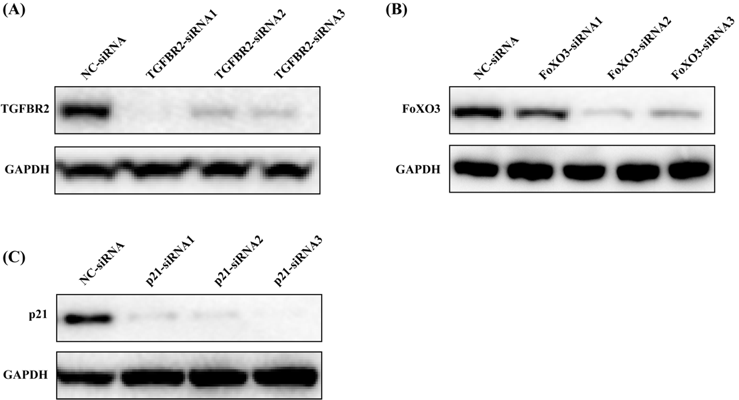


Figure. S3. The knockdown efficiency of by siRNA targeting TGFBR2, FoXO3 and p21 respectively. (A) Western blotting of TGFBR2 in PC-3 cells transfected NC-siRNA, TGFBR2-siRNA1, TGFBR2-siRNA2 and TGFBR2-siRNA3, respectively. (B) Western blotting of FoXO3 in PC-3 cells transfected NC-siRNA, FoXO3-siRNA1, FoXO3-siRNA2, and FoXO3-siRNA3, respectively. (C) Western blotting of p21 in PC-3 cells transfected NC-siRNA, p21-siRNA1, p21-siRNA2, and p21-siRNA3, respectively.

Figure. S4.


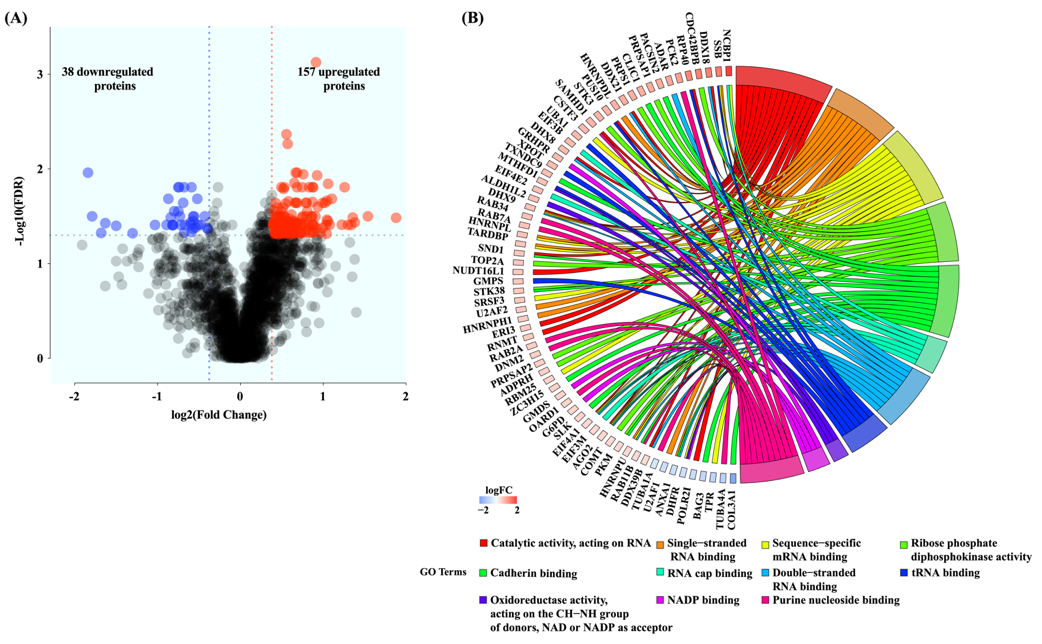


Figure. S4 The differentially expressed proteins of undefined-secreted proteins. (A) Volcano plots presented differentially expressed proteins in undefined-secreted proteins. The red and blue dots represented up-regulated and down-regulated proteins, respectively. (B) The molecular function analysis of differentially expressed proteins in undefined-secreted proteins. Proteins were linked with their assigned GO terms via ribbons. Red-to-blue rectangles coded next to selected proteins with their log_2_(fold change) (logFC). GO terms of molecular function were arranged from top to bottom according to their significances.

Figure. S5


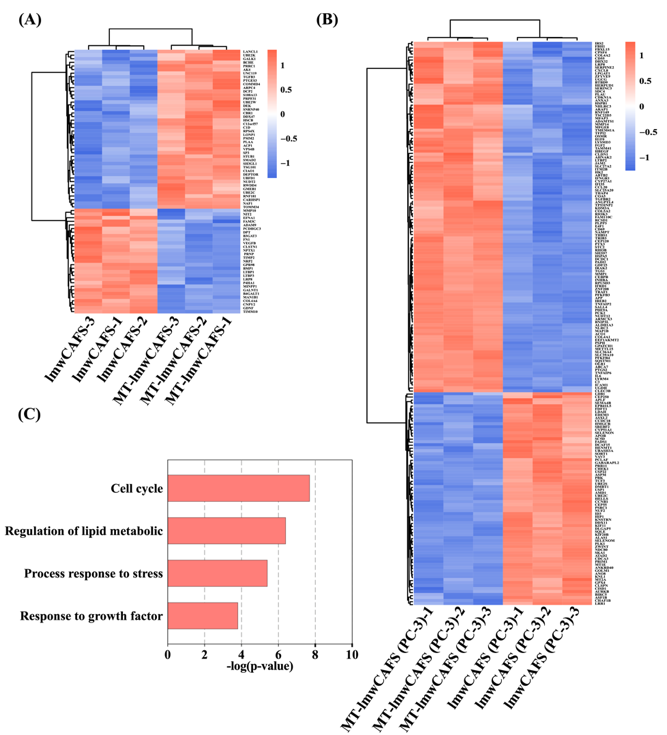


Figure. S5 The differentially expressed proteins in genuine-secreted proteins and lmwCAFS treated PC-3 cells/MT-lmwCAFS treated PC-3 cells. (A) The heatmap of differential expression proteins in lmwCAFS and MT-lmwCAFS. (B) The heatmap of differential expression proteins in lmwCAFS treated PC-3 cells and MT-lmwCAFS treated PC-3 cells. (C) The biological process analysis of the differentially expressed proteins in mwCAFS treated PC-3 cells and MT-lmwCAFS treated PC-3 cells.
