# Supplementary material for: Proteomics reveals the function reverse of MPSSS‐treated prostate cancer‐associated fibroblasts to suppress PC‐3 cell viability via the FoxO pathway
Source: Cancer Med. 2021 Mar 11;10(7):2509–22. doi: 10.1002/cam4.3825 (PMC7982613; doi:10.1002/cam4.3825)
Supplement: Supplementary file 2 — Table S1 [file CAM4-10-2509-s003.docx]

**Table S1. P****rimer sequences for RT-PCR analysis of p21 and GAPDH.**

| **Names** | **Forward primer** | **Reverse primer** |
| --- | --- | --- |
| p21 | 5’- TGTCCGTCAGAACCCATGC-3’ | 5’- AAAGTCGAAGTTCCATCGCTC-3’ |
| GAPDH | 5’-GGAGCGAGATCCCTCCAAAAT-3’ | 5’-GGCTGTTGTCATACTTCTCATGG-3’ |
