# Supplementary material for: Proteomics reveals the function reverse of MPSSS‐treated prostate cancer‐associated fibroblasts to suppress PC‐3 cell viability via the FoxO pathway
Source: Cancer Med. 2021 Mar 11;10(7):2509–22. doi: 10.1002/cam4.3825 (PMC7982613; doi:10.1002/cam4.3825)
Supplement: Supplementary file 3 — Table S2 [file CAM4-10-2509-s002.docx]

**Table S2. The siRNA sequences for FoXO3, TGFBR2, p21, and the negative control**

| siRNA | | Sequences |
| --- | --- | --- |
| Negative Control | | 5’- UUCUCCGAACGUGUCACGUTT-3’ |
|  |  | 5’- ACGUGACACGUUCGGAGAATT-3’ |
| FoXO3 | siRNA1 | 5’-GCUGUCUCCAUGGACAAUATT-3’ |
|  |  | 5’-UAUUGUCCAUGGAGACAGCTT-3’ |
|  | siRNA2 | 5’-GCUCACUUCGGACUCACUUTT-3’ |
|  |  | 5’-AAGUGAGUCCGAAGUGAGCTT-3’ |
|  | siRNA3 | 5’-CCUCAUCUCCACACAGAAUTT-3’ |
|  |  | 5’-AUUCUGUGUGGAGAUGAGGTT-3’ |
| P21 | siRNA1 | 5’-GAUGGAACUUCGACUUUGUTT-3’ |
|  |  | 5’-ACAAAGUCGAAGUUCCAUCTT-3’ |
|  | siRNA2 | 5’-CCUCUGGCAUUAGAAUUAUTT-3’ |
|  |  | 5’-AUAAUUCUAAUGCCAGAGGTT-3’ |
|  | siRNA3 | 5’-CAGGCGGUUAUGAAAUUCATT-3’ |
|  |  | 5’-UGAAUUUCAUAACCGCCUGTT-3’ |
| TGFBR2 | siRNA1 | 5’-GACCUCAAGAGCUCCAAUATT-3’ |
|  |  | 5’- UAUUGGAGCUCUUGAGGUCTT-3’ |
|  | siRNA2 | 5’- UCCUGCAUGAGCAACUGCATT -3’ |
|  |  | 5’- UGCAGUUGCUCAUGCAGGATT-3’ |
|  | siRNA3 | 5’- GCUUCUCCAAAGUGCAUUATT -3’ |
|  |  | 5’- UAAUGCACUUUGGAGAAGCTT-3’ |
